# Polymyxin B in Patients With Renal Impairment: Is It Necessary to Adjust Dose?

**DOI:** 10.3389/fphar.2022.955633

**Published:** 2022-06-28

**Authors:** Ruifang Nie, Dejun Li, Peng Wang, Genquan Yan, Bing Leng

**Affiliations:** ^1^ Department of Pharmacy, Shandong Provincial Hospital Affiliated to Shandong First Medical University, Jinan, China; ^2^ Department of Intensive Care Unit, Shandong Provincial Hospital Affiliated to Shandong First Medical University, Jinan, China

**Keywords:** polymyxin B, antibiotics, renal impairment, dose adjustment, creatinine clearance

## Introduction

Polymyxins (colistin and polymyxin B) are lipopeptide antibiotics that were available clinically in the late 1950s but fell out of favor in the 1970s owing to their potential nephrotoxicity and the availability of less toxic antibiotics. However, they were reintroduced clinically in the last decade because of their high sensitivity against multidrug-resistant Gram-negative bacteria (Poirel et al., 2017; Zakuan and Suresh, 2018). Colistin and polymyxin B have similar chemical structures and antibacterial activity *in vitro*, but different metabolic pathways. As a prodrug, 60%–70% of colistin is excreted by the kidneys, and it is converted into its active form in the urinary tract (Couet et al., 2011; Tran et al., 2016). Thus, a consensus was reached to adjust the colistin dose on the basis of kidney function. For polymyxin B, which is administered in its pharmacologically active form and mainly eliminated by non-renal pathways, the dosing regimen in patients with renal impairment remains controversial.

### What is the Optimal Polymyxin B Dose for Patients With Renal Impairment in the Drug Label and Latest Guidelines?

The dosing strategy recommended in the current United States Food and Drug Association-approved label is 15,000 to 25,000 units/kg/day (1.5–2.5 mg/kg/d) for individuals with normal kidney function, and a reduced dose for individuals with renal impairment is recommended, although no relevant research evidence was provided (U.S. Food and Drug Administration, 2012). However, the International Consensus Guidelines for the Optimal Use of the Polymyxins did not suggest a dose adjustment (Tsuji et al., 2019). On the basis of research conducted by Zavascki et al., Kwa et al., Sandri et al., Thamlikitkul et al., and Nelson et al. (Table 1, Entries 1–5), the guidelines recommend a loading dose of 2.0–2.5 mg/kg and a maintenance dose of 1.25–1.5 mg/kg every 12 h, regardless of the patient’s renal function.

**TABLE 1:** Summary of related studies.

Entry	Study	Research Method	Patients Included	Polymyxin B Dose	View
1	Zavascki et al. (2008)	PK study	8 critically ill patients (CrCl<246 ml/min)	1.0 ± 0.3 mg/kg every 12/48 h	No dose adjustment
2	Kwa et al. (2011)	PK study	1 patient (CrCl: 18 ml/min), without dialysis	loading dose: 50 mg; maintenance dose: 75 mg every 24 h	
3	Sandri et al. (2013)	population PK model/Monte Carlo simulations	24 critically ill patients (CrCl: 10–143 ml/min), of which 2 received CVVHD	0.45–3.38 mg/kg/day	
4	Thamlikitkul et al. (2016)	PK study	5 patients with normal renal function (CrCl: 90.0 ± 12.5 ml/min); 14 with renal insufficiency (CrCl: 40.8 ± 21.8 ml/min). all without RRT	normal renal function: 2.2 ± 0.2 mg/kg/day; renal insufficiency: 1.9 ± 0.3 mg/kg/d	
5	Nelson et al. (2015)	retrospective study	151 patients with blood infections, of which 42 received RRT	the median dosage:1.3 mg/kg/day (30 < CrCl <50 ml/min: 1.5 mg/kg/day; CrCl <30 ml/min or receiving RRT:1–1.5 mg/kg every 2–3 days)	
6	Manchandani et al. (2018)	population PK/maximum likelihood expectation maximization approach	35 adult patients (CrCl: 67 ± 42 ml/min)	2.1 ± 0.4 mg/kg/day	
7	Wang et al. (2021)	population PK models	37 patients with normal renal function (CrCl: 81.6–315.2 ml/min); 33 with renal insufficiency (CrCl: 15.6–77.6 ml/min). all without RRT	2.0 ± 0.5 mg/kg/day	
8	Maniara et al. (2020)	retrospective chart review	54 patients with non-AKI (CrCl<80 ml/min), without RRT	non-adjusted: 1.5–2.5 mg/kg/d; adjusted: <1.5 mg/kg/day	
9	Peyko and Cohen, (2018)	prospective cohort and retrospective cohort	22 patients with no renal dose adjustments (CrCl: 32.5 ± 15.70 ml/min); 20 patients with renal dose adjustments (CrCl: 41.5 ± 10.05 ml/min). all without hemodialysis	non-adjusted: 1.90 ± 0.317 mg/kg/day; adjusted: 1.13 ± 0.278 mg/kg/day	
10	Yu et al. (2020)	retrospective examination	5 patients with renal impairment receiving supportive CRRT (average CrCl: 21.6 ml/min)	1.33–2.00 mg/kg/day	Dose adjustment
11	Yu et al. (2021)	population PK model/Monte Carlo simulations	32 adult patients with varying renal function (CrCl: 86.58 ± 53.00 ml/min), without RRT	2.26 ± 0.49 mg/kg/day	
12	Li et al. (2021)	population PK model/Monte Carlo simulations	50 renal transplant patients (CrCl:4.29–90.7 ml/min), of which 11 had CRRT	most common dose was 40 and 50 mg every 12 h	
13	Wu et al. (2021)	population PK model/Monte Carlo simulation	37 patients with normal renal function (CrCl:81.6–315.2 ml/min); 33 with renal insufficiency (CrCl:15.6–77.6 ml/min). all without RRT	2.0 ± 0.5 mg/kg/day	

PK, pharmacokinetic; CrCl, creatinine clearance; CVVHD, continuous venovenous hemodialysis; RRT, renal replacement therapy; CRRT, continuous renal replacement therapy; AKI, acute renal injury.

Zavascki et al. (Zavascki et al., 2008) conducted a study that enrolled 8 critically ill patients who received 1.0 ± 0.3 mg/kg polymyxin B every 12/48 h in 2008. They found that only 0.04–0.86% of the dose was recovered in the urine in its unchanged form, indicating that polymyxin B is eliminated mainly by non-renal pathways (Table 1, Entry 1). Subsequent studies provided further evidence to support this conclusion. In 2011, Kwa et al. (Kwa et al., 2011) provided a case that a patient weighed 50 kg with a baseline serum creatinine level of 3.5 mg/dl (estimated creatinine clearance, 18 ml/min), was given intermittent intravenous infusions polymyxin B with a loading dose of 50 mg and maintenance dose of 75 mg every 24 h. The polymyxin B half-life was calculated as 11.5 h, which is similar to data that were previously observed in patients with normal renal function (Table 1, Entry 2). In 2013, Sandri et al. (Sandri et al., 2013) conducted a population pharmacokinetic analysis and Monte Carlo simulations on 24 critically ill patients using blood and urine samples. The median urinary recovery of polymyxin B was 4.04%, and the total body clearance of polymyxin B did not show any relationship with creatinine clearance (r^2^ = 0.008; Table 1, Entry 3). Thamlikitkul et al. (Thamlikitkul et al., 2016) conducted a study involving 19 adult patients who received intravenous polymyxin B (2.2 ± 0.2 mg/kg/day or 1.9 ± 0.3 mg/kg/d) in 2016, which proved polymyxin B exposures in patients with normal and impaired renal function were comparable. However, patients in the renal impairment group had lower dose levels than those with normal renal function, even though with no statistically significant difference (*p* = 0.08; Table 1, Entry 4). Nelson et al. (Nelson et al., 2015) also conducted a retrospective study to evaluate the relationship between the polymyxin B dose and clinical outcomes. Based on the results that 82% of patients receiving polymyxin B doses <1.3 mg/kg/day had baseline renal impairment and that the doses were significantly associated with 30-days mortality, Nelson et al. found a reduction in polymyxin B in patients with renal insufficiency may result in decreased efficacy and increased 30-days mortality. (Table 1, Entry 5).

On the basis of the above studies, the International Consensus Guidelines for the Optimal Use of the Polymyxins concluded that the polymyxin B dose should not be adjusted based on renal function. However, we found that the polymyxin B dose in most of the referenced studies were all below 2.5 mg/kg/d, and it is not reasonable to use the findings of these low-dose studies to extrapolate conventional doses (2.5–3.0 mg/kg/d). Therefore, this evidence seems to be insufficient to support the guidelines’ recommendations.

### What are Recent Recommendations in the Latest Research?

Some recent studies provided new evidence for not adjusting the dose (Table 1, Entries 6–9). In 2018, Manchandani et al. (Manchandani et al., 2018) conducted a population pharmacokinetic study of polymyxin B including 35 adult patients to assess factors influencing the pharmacokinetic variability. Although they found that creatinine clearance was an important covariate of polymyxin B clearance (r^2^ < 0.3), the magnitude of the association was considered clinically insignificant (Table 1, Entry 6). Wang et al. (Wang et al., 2021) subsequently conducted a study that enrolled 37 patients with normal renal function and 33 patients with renal insufficiency in 2021, and they came to a similar conclusion. The simulated AUC_ss,24h_ values for patients with normal renal function were lower than those for patients with renal insufficiency, but this difference was not statistically significant (Table 1, Entry 7). In 2020, Maniara et al. (Maniara et al., 2020) divided patients with renal impairment into a renally adjusted arm (polymyxin B dose adjusted on the basis of renal function) and a non-renally adjusted arm, and they found that the incidence of nephrotoxicity and mortality were higher in the renally adjusted arm compared with those in the non-renally adjusted arm (21.7 *vs*. 6.5% and 16 *vs*. 14 days, respectively) (Table 1, Entry 8). These results caused some confusion, so we re-analyzed the data and found that there was no statistically significant difference in the incidence of neurotoxicity (*p* = 0.386) and mortality (*p* = 0.206) between the two groups. The following research by Peyko et al. (Peyko and Cohen, 2020) also supported this conclusion. They found that there was no difference between the two groups in the incidence of acute kidney injury at day 7, occurrence of microbiological cure, clinical cure, and 30-days mortality (Table 1, Entry 9).

There are also some studies that supported the view that the polymyxin B dose should be adjusted in renal impairment patients (Table 1, Entries 10–13). In 2020, Yu et al. (Yu et al., 2020) demonstrated that a relatively low dose of polymyxin B (a 100-mg daily dose for patients weighing between 50 and 75 kg) could control pulmonary infections in patients with renal impairment (Table 1, Entry 10). In 2021, Yu et al. (Yu et al., 2021) developed a population pharmacokinetic model of polymyxin B and Monte Carlo simulations on the basis of a retrospective study in 32 adult patients with varying renal function. Results demonstrated that creatinine clearance was the significant covariate in polymyxin clearance (normal value, 1.59 L/h; between-subject variability, 13%), and the polymyxin B dose should be adjusted on the basis of creatinine clearance (Table 1, Entry 11). For example, the dose should decrease 33% for patients with moderate renal insufficiency (30 ≤ creatinine clearance <60 ml/min). To optimize the dosing strategy and reduce the risk of toxicity, Li et al. (Li et al., 2021) developed a population pharmacokinetic model of polymyxin B for Chinese renal transplant patients, showing that renal function plays a key role in polymyxin B clearance, and a 75-mg loading dose with a 50-mg maintenance dose was an optimal dosing strategy for patients with renal impairments (Table 1, Entry 12). Wu et al. (Wu et al., 2021) also conducted a Monte Carlo simulation using data from Wang et al.’s study (Wang et al., 2021), but their study presented different conclusions. Wu believed that the polymyxin B dose needed to be adjusted, and they provided a recommendation for Chinese patients. Their recommendation was that a loading dose of 2.5 mg/kg should be administered regardless of renal function, a maintenance dose of 60 mg should be administered every 12 h for patients with renal impairment, and a dose of 1.25 mg/kg should be administered for patients with normal renal function (Table 1, Entry 13).

### What Is Our Opinion?

There are many studies on the polymyxin B dose in patients with renal impairment, but because of the design limitations and conflicting results, it is not currently possible to draw a consensus conclusion. Thus, there is an urgent need for studies with a more reasonable dose range.

Currently, most published pharmacokinetic studies agree that creatinine clearance has an effect on polymyxin B clearance, but the magnitude of the effect remains controversial. Whether it is necessary to adjust the dose and how to adjust it are important future research areas.

In the other hand, clinical observational studies confirmed that patient groups with or without a dose adjustment showed similar efficacy and safety indicators. Since the adjusted group is more economical under the condition of ensuring sufficient effectiveness, we think a dose adjustment is worth recommending. However, how to adjust the dose depends on the severity of the renal damage. We are currently collecting data from patient cases and blood samples to explore a dose adjustment regimen that can provide both efficacy and safety.
